# The impact of facility and staff characteristics on infection control of COVID‐19: Perceptions of elder care home managers in Stockholm, Sweden

**DOI:** 10.1002/nop2.2169

**Published:** 2024-05-23

**Authors:** Ann E. M. Liljas, Lenke P. Morath, Bo Burström, Pär Schön, Janne Agerholm

**Affiliations:** ^1^ Department of Global Public Health Karolinska Institutet Solna Sweden

**Keywords:** ageing, infectious disease outbreaks, management, nursing homes, older adults, organisational characteristics, pandemic, qualitative study, residential long‐term care facilities

## Abstract

**Aim:**

To examine the perceptions of managers of elder care homes on the impact of facility and staff characteristics on infection control of COVID‐19.

**Design:**

Case study.

**Methods:**

Six purposively sampled care home managers in the city of Stockholm were interviewed. Through content analysis, three categories and nine subcategories were identified.

**Results:**

According to the interviewed care home managers, a home‐like environment that allows for isolation of residents and possibilities for staff to get changed and store personal protective equipment outside each resident's room was considered ideal. Experienced employees were reported as invaluable when facing an infectious outbreak. A mix of permanent and temporary staff was considered essential although some thought that temporary staff who work in multiple care homes might negatively influence the spread of infection. Language barriers among staff were considered an obstacle when trying to disseminate information.

## 1 BACKGROUND

Residing in a care home for older adults (also known as nursing homes or residential long‐term care facilities) involves close contact with staff and other residents, making it difficult to practice social distancing. In many countries, residents also share rooms (Stern & Klein, 2020). Modern care homes are often designed to bring people together and provide a home‐like atmosphere, however, during infectious disease outbreaks, such design challenges the possibility of isolating residents and conducting cohort care to prevent spread of infection (McClean et al., 2012; Mo & Shi, 2020). The ongoing COVID‐19 pandemic has disproportionately affected older populations, especially older adults with multi‐morbidity who reside in care homes, due to challenges in preventing and controlling transmission of the virus (Anand et al., 2021). Additionally, the role of care home staff in the spread of infection including staff working at multiple facilities has been debated (Ouslander & Grabowski, 2020). Research on staff characteristics in relation to outbreaks of infectious diseases in care homes is, however, scant (Assariparambil et al., 2021). Our recent systematic review on the potential impact of staff and facility characteristics on infectious disease outbreaks in care homes suggests that staff compartmentalising (i.e. dividing staff between units at the care home) may lower the risk of an outbreak, whereas staff residing in highly infected areas may be associated with greater risk of an outbreak (Liljas et al., 2022). Due to inconsistency in the findings of the studies included in the systematic review, the impact of facility design, use of temporary staff and the number of nursing aide hours per resident remains unclear. Nonetheless, in comparison to age‐related health decline in older residents, staff and facility characteristics are considered modifiable and therefore of interest to improve infection control (Harrington et al., 2020; Wang, 2020). For this study, care home managers were targeted as they have insight into the staffing situation and physical building. This study aims to examine the perceptions of managers of care homes for older adults on the impact of facility and staff characteristics on infection control of COVID‐19.

### 1.1 Care homes in Sweden

This study was undertaken in Stockholm, Sweden. In comparison to other Swedish cities and the other Nordic capitals, Stockholm was particularly hard hit by the pandemic where care home residents accounted for up to 50% of all deaths at the time of data collection (European Centre for Disease Prevention and Control, 2020; Szebehely, 2020).

Care homes in Sweden provide social care by nursing aides (24/7) and healthcare by nurses who are on‐site daily. At night and the weekends, nursing aides provide the care needed with the opportunity to contact nurses on‐call. Care homes are funded through local taxation and provided by the municipality or private companies (publicly funded). In Sweden, older adults tend to live in their own private homes with home care provided multiple times per day until such support is no longer sufficient to independently undertake everyday activities. Subsequently, by the time older adults move into a care home, they tend to have extensive care needs (Stern & Klein, 2020).

In Swedish care homes, residents have their own home‐like apartment in the form of a bedroom with ensuite bathroom by standard (Swedish Association of Local Authorities and Regions, 2020). Most care homes in Sweden do neither have the necessary medical equipment required to provide oxygen and intravenous drips nor access to medically trained staff including nurses 24/7 (Johansson & Schön, 2021). This and other structural shortcomings of the care home sector such as fragmentation, insufficient regulations and inadequate staffing were highlighted as explanations as to why Swedish care homes were unprepared and ill‐equipped for the COVID‐19 pandemic (Coronakommissionen, 2020). Collaboration between healthcare and social care is complicated in Sweden due to its highly decentralised institutional structure where 290 municipalities are responsible for social care, whereas the 21 regions are responsible for healthcare. Yet, management, coordination, efficiency and responsibility are fundamental components of care homes that relate to both healthcare and social care (Schön & Fritzell, 2021). Increased collaboration between health and social care has the potential to improve care including infection control for the residents (Bäck & Calltorp, 2015).

## 2 METHODS

### 2.1 Study design

This is an exploratory–descriptive qualitative research study with a case study design. The case study design allows for knowledge to be gained about a specific topic, for example, facility and staff characteristics on COVID‐19 infection control, in a specific setting, that is, care homes. The case study design also enables perspectives of the wider social and political environment that has shaped the issue studied (Doolin, 1998). The COREQ checklist for reporting on qualitative research has been used (Data S1).

### 2.2 Study setting and study participants

The study targeted managers of care homes located in the city of Stockholm. Stockholm was chosen as it was particularly hard hit by the pandemic. Thirteen care homes were purposively sampled from the local government's website (https://aldreomsorg.stockholm/hitta‐vard‐och‐omsorgsboende/) listing all 151 care homes (31 public and 120 private care homes) across Stockholm to capture diversity in facility size and ownership status. The care home managers were telephoned and informed about the study and its aim. Nine of them were scheduled for an interview, however, three dropped out due to time constraints. Two of the remaining six managers redirected the request to a nurse with management responsibility considered to have the same or greater insight as themselves to the efforts of infection prevention at the facility. The nurses were deemed relevant to include as participants and hereinafter all participants are referred to as informants or care home managers.

Following the initial telephone call, all participants were emailed information about the study. Informed consent was given by all participants prior to data collection. The study has received research ethics committee approval (2020‐05850).

### 2.3 Data collection

An interview topic guide based on existing literature and a multidisciplinary team discussion was developed. The first part of the interview covered characteristics of the care home (e.g. number of apartments, number of employees and ownership status); information which was found online prior to the interview and confirmed by the informant at the beginning of the interview. The second part primarily consisted of open‐ended questions about actions taken to prevent COVID‐19 outbreaks, the impact of the facility design and the impact of staffing on COVID‐19 outbreak prevention and control. The interview topic was tested in a pilot interview, resulting in a few minor changes to the wording and order of some of the questions. The semi‐structured interviews were conducted one to one by female researcher LM who is a medical student of younger age than the participants, with a background as a journalist and experience of interviewing people. Researcher LM also had a pre‐understanding and assumptions of care homes for older adults from her previous work experiences as a nursing aide in several care homes (not at any of the care homes approached). The care home managers approached and interviewed were not known by any of the researchers. All questions were asked at each interview albeit not always in the order presented in the topic guide (Appendix A).

Due to the ongoing COVID‐19 pandemic, the interviews were conducted by telephone, or video call using Zoom. The interviews were conducted between 25th February and 17th March, 2021, based on each participant's availability and preference. The interviews lasted for about 1 h, were audio recorded using a Zoom H2n Handy Recorder and transcribed verbatim by the interviewer. Identifiable information was anonymised. The interviewer took field notes after each interview. The audio recordings and interview transcripts were uploaded and stored on a server at the university where the researchers are affiliated, using a two‐factor authentication.

### 2.4 Data analysis

For the data analysis, Graneheim and Lundman's content analysis for qualitative health research was applied, commonly used for the analysis of qualitative health research and suitable for analysis of entire interviews (Graneheim & Lundman, 2004). In accordance with this content analysis approach, two researchers (LM and AL (postdoctoral)) read the transcribed interviews thoroughly multiple times to gain a deep understanding of the data. For the first two transcribed interviews, the researchers worked together dividing the text into meaning units. Each meaning unit was condensed, abstracted and labelled with a code. Occasionally, multiple meaning units corresponded to the same code. The researchers repeatedly discussed and revised the coding and eventually agreed upon 26 codes. Next, researcher LM coded all data of the remaining transcripts. The two researchers combined the codes into subcategories which were grouped into three main categories (Graneheim & Lundman, 2004). Then, the underlying meaning of the categories was formulated into a theme. The analysis of the transcripts was conducted by the two researchers using MS Excel software. Interpretations of the results were drawn by the entire team. The theme, categories, subcategories and quotes were then translated into English. The choice of words and synonyms were discussed within the team to make the translations as accurate as possible. Participants have been given the opportunity to read and comment on the manuscript.

### 2.5 Rigour and trustworthiness

The rigour of the study was ensured by being transparent in the reporting of the research process step by step. Multiple approaches were applied to ensure trustworthiness including credibility, dependability, confirmability and transferability (Lincoln & Guba, 1986). Credibility was sought by using a well‐known research method and analytical approach including taking field notes at the end of each interview and two researchers organising the text in the interview transcriptions into meaning units. Dependability was sought by having two researchers coding and by providing detailed information about the research process enabling the reader to assess the research practice. Confirmability was ensured by supporting the results with quotes from the participants, through member checking, that is, letting the participants read and comment on the manuscript, and by involving at least two researchers in all steps of the analytical parts, reducing the risks of analysis bias and allowing the results to be interpreted and analysed from more than one perspective. Transferability to other contexts was sought by describing the phenomenon studied, the setting and the participants.

## 3 RESULTS

### 3.1 Characteristics of participating care homes

Informants from six care homes were interviewed. The characteristics of these care homes are presented in Table 1 showing that most care homes operated in one building with multiple floors and were privately owned. The size of the care homes referred to the number of apartments (with typically one bed per apartment) and ranged from two smaller care homes (<50 apartments), three middle‐sized care homes (50–100 apartments) to one large care home (>100 apartments). The proportion of temporary hired employees (employed by the hour) was estimated by the informant and reported as equal to or below a third of the total workforce in all care homes.

**TABLE 1:** Characteristics of the participating care homes.

	Informant's work position	Ownership status	Number of apartments	Number of employees	Proportion of temporary employees	Number of buildings	Number of floors
Informant 1	Nurse (with management responsibilities)	Municipality	>100	>150	<1/3	2	7 resp. 9
Informant 2	Manager	Private	<50	<100	<1/3	1	4
Informant 3	Manager	Private	<50	<100	≈1/3	1	1
Informant 4	Nurse (with management responsibilities)	Private	50–100	<100	<1/3	1	3
Informant 5	Manager	Municipality	50–100	>150	≈1/3	1	6
Informant 6	Manager	Private	50–100	>150	≈1/3	1	6

The categories and subcategories identified are listed in Table 2 and presented below by category, starting with ‘The homelike facility’, followed by ‘Challenges related to the workforce’ and lastly, ‘Attempts of infection control’.

**TABLE 2:** Study theme, categories and sub‐categories.

Theme	Efforts to provide infection‐free care under suboptimal conditions		
Categories	The home‐like facility	Challenges related to the workforce	Attempts of infection control
Sub‐categories	A facility design that reflects the purpose of care homes Challenges in isolating residents with dementia The impact of facility size and physical environment	Absence at the beginning of the pandemic The dissemination of information The importance of experienced employees Employees' living and work situation	Actions to mitigate spread of infection Actions to reduce social contact

### 3.2 The home‐like facility

The informants reported that the COVID‐19 pandemic had made them reflect on the facility design of the care home. Their perceptions of the facility design are presented based on the three subcategories ‘A facility design that reflects the purpose of care homes’, ‘Challenges in isolating residents with dementia’ and ‘The impact of facility size and physical environment’.

#### 3.2.1 A facility design that reflects the purpose of care homes

All informants described how they had struggled to provide healthcare during the pandemic as the care home facilities offer home‐like environments with common areas for dining and social activities.

We discovered that our facility is not designed to provide care during a pandemic, so to speak. Because this is [built to feel like] a home. (Informant 1)

The informants thought that changing the facility design would not necessarily be a good thing. They reflected upon whether the care home should mainly be a home or a facility that provides medical healthcare of which most stressed the importance of allowing the care home to primarily remain a home.

Obviously, one could build the facility to become more like a hospital ward. Certainly, that would be of use in situations like this. But would that be a place where older adults want to live? (Informant 2)

Regarding differences in the facility design between care homes and hospitals, the informants thought they would benefit from having a smaller outer room known as an ante‐room, before entering the resident's apartment. The wish for an ante‐room re‐occurred in the interviews and was directly related to the unmet need to find a suitable place to store personal protective equipment (PPE).

I believe that if it had been for example a hospital, there would have been ante‐rooms… (Informant 2)

Then you have the aspect of having no ante‐room and no place to store PPE etcetera. Since this is the home of the residents you can't place PPE wherever you like. Those kinds of difficulties we faced. (Informant 1)

Another issue reported by some of the informants was the lack of areas designated to hygiene. The advantages of having a separate washing machine for each resident were given as an example.

I think, when it comes to aspects of hygiene, hospitals have a bit more of that, even if there have been changes to their buildings too. The hygienic aspect has somewhat got lost in the re‐structuring of the care home facility [towards a homelike environment]. (Informant 5)

#### 3.2.2 Challenges in isolating residents with dementia

All informants reported facing challenges isolating residents with dementia in the event of an infectious outbreak.

I mean, we have no right to lock them up, we can't lock the doors to our residents. And if they want to walk out [they can do so]. (Informant 3)

The informants reported that the difficulties of isolating a person with dementia sometimes resulted in the isolation of entire units rather than just isolating single residents in their own apartments.

If [the infected] residents were ill and bedbound, it was easier to organise cohort care. In that case two members of staff were appointed to the rooms with infected residents only. But when the infected residents were more alert and wandered, we had to isolate the unit as a whole. (Informant 1)

The importance of having the possibility to isolate small units was brought up by nearly all informants. This was considered particularly important when caring for residents with dementia since isolating an entire unit was sometimes the only solution.

Obviously it would have been helpful to be able to cut‐off more [areas], that's impossible to get around. (Informant 2)

#### 3.2.3 The impact of facility size and physical environment

Informants based their responses to the questions about facility size and design on what the care home they operate looks like. All considered that the floor plan influenced the way the facility organised its COVID‐19 strategy. For instance, not having to walk through units to get from A to B or to have an extra entrance was considered beneficial. Floor plans that divide units from each other, having individual balconies for each apartment, spacious rooms and outdoor areas were also highly valued facility characteristics.

… on every floor, we have a big glass balcony with infrared heating and so on, and then, we have a courtyard. So, that has been really good. (Informant 6)

I believe that the best thing is to have space, quite a lot of space in the facility so that it isn't too tight and crowded. (Informant 4)

Most of the informants also thought that a care home with a smaller number of residents is preferable in terms of infection prevention and control.

Because of the [large] size, there are more persons around here. That affects the spread of the infection. If one had had a small care home with 20 residents, maybe COVID‐19 wouldn't have entered the facility at all. (Informant 1)

### 3.3 Challenges related to the workforce

All informants discussed how the pandemic affected staff as well as how the staffing situation impacted their possibilities to handle the challenges of the pandemic. Their thoughts are presented below according to the four subcategories: absence at the beginning of the pandemic, employees' living and work situation, the importance of experienced personnel and the dissemination of information.

#### 3.3.1 Absence at the beginning of the pandemic

Almost all informants reported the beginning of the pandemic to be very burdensome as the care homes were challenged by a significant absence of permanent staff. The high rates of sick leave were regarded as a consequence of the recommendation to stay home even with the slightest symptoms. However, many informants also mentioned how fear of the new disease contributed to staff staying home.

Really, it was both [sickness and fear]. People were scared. [And] there were also those who were sick for sure. (Informant 5)

#### 3.3.2 Employees' living and work situation

Some informants thought that employees' living situations might have influenced whether they decided to go to work or not during the pandemic. Examples included staff being particularly anxious about getting infected if they had older close relatives and family members who depended on them in their everyday lives. The informants reported that they have been very strict in sending home symptomatic staff and that asymptomatic staff were just as likely to be permanently employed as employed by the hour. Hiring employees by the hour allowed for a larger workforce and enabled stricter compartmentalisation of staff between units, and overall temporary employees were considered a fundamental contribution to the workforce both during the pandemic and ordinary circumstances.

No matter what, we have to have employees hired by the hour since people have all kinds of reasons for being absent. And we have to be able to make changes in the staffing at each unit. I mean, if we have a lot of empty apartments on one floor, we have to have a smooth approach to cut down on staffing, it isn't possible any other way. (Informant 2)

Some informants thought that employees hired by the hour might be more likely to spread the infection as they are more likely to work at multiple care homes compared to permanent staff.

I can imagine that if you are employed by the hour you are more dependent on your working shifts and that might somewhat influence whether you acknowledge that you're feeling a bit seedy and stay home. It could be like that as they are [financially] dependent on working all their shifts. (Informant 5)

#### 3.3.3 The importance of experienced employees

Many informants reported that they had hired staff temporarily to cover for employees in sickness absence during the pandemic. While most employees were replaced by staff who undertook extra work shifts or temporarily hired social care workers to cover for those absent, replacing trained and experienced staff with people from other disciplines with no experience from the care sector made the situation particularly challenging and stressful. Mainly employees' expertise was emphasised and considered invaluable by the informants. Although the COVID‐19 pandemic was an extraordinary situation, employees who had been at the care homes for many years had experienced other infectious disease outbreaks such as calicivirus and gastroenteritis.

…the expertise that our staff has, [they know] how difficult it is to care for a person with dementia. It is rocket science, but people don't understand that. People think that any person in the street can do the job. But they can't. (Informant 3)

#### 3.3.4 Dissemination of information

In the interviews, the informants stressed how the beginning of the pandemic was characterised by great insecurity regarding the virus and a constant stream of new information.

Nobody knew anything and such lack of knowledge was the worst, for all of us. … The most important thing was to remain calm and try to inform staff about the virus even though we didn't know much ourselves. (Informant 4)

The informants reported on different strategies to pass on information to staff.

In the beginning, there was new information every day, almost every hour. So we introduced a “corona file” with printed copies of up‐to‐date information available in the staff room. (Informant 3)

We definitely tried to keep employees informed. We really lavished information upon our employees, both in written and orally. (Informant 6)

The informants thought that the constant changes to the information and recommendations were further complicated by language barriers. Such barriers referred to both differences in languages and unfamiliarity of reading written text in Swedish.

It has been a challenge to reach out to employees with information. … For one thing, [if] you are not used to read a lot of text, you won't do [read] it. And then you might not have Swedish as your mother tongue. (Informant 3)

### 3.4 Attempts of infection control

Attempts to control the infection spread were reported by all informants. Their experiences are presented in two subcategories: actions to mitigate spread of infection and actions to reduce social contact.

#### 3.4.1 Actions to mitigate spread of infection

To maintain resident safety, all informants reported that they either had implemented a new organisation or re‐organised the way of working.

We feel that we have established a way of working so that we immediately initiate and perform all of the arrangements that needs to be initiated to mitigate spread of infection. We have also re‐furnished the dining room and the living room so that residents don't sit too close to each other. (Informant 5)

Many of the informants stressed the importance of basic hygiene routines and use of PPE, although PPE was not always available at the beginning of the pandemic.

…That everyone follows basic hygiene routines and use the PPE that we have got and have access to. (Informant 4)

This huge discussion about facemasks versus no facemasks. In the elderly care, we didn't have any facemasks. But the things you saw on TV from the hospitals… Well, they basically had gas masks whenever there was a risk of infection. You had to try to explain that [to staff], and that wasn't particularly easy. (Informant 1)

#### 3.4.2 Actions to reduce social contact

All informants discussed the importance of and strategies to reduce and track social contacts in the facility.

Employees hired by the hour who could go between all of the floors had to be limited to a maximum of two floors in order to not expose all floors to potential infection. (Informant 6)

The informants further reported on the importance of the visiting ban followed by organising for safe visits, even though they noticed that it sometimes affected everyday life of the residents negatively. All informants also emphasised the importance of providing cohort care, meaning that staff were strictly divided between infected and non‐infected residents.

Every time we had the slightest suspicion [of a potential infectious outbreak], we immediately started providing cohort care. At the time of the smaller infection outbreak that we had in the autumn [2020], we arranged for cohort care teams who only cared for the infected. (Informant 6)

## 4 DISCUSSION

### 4.1 Summary of results

This study provides insight into six Stockholm‐based care home managers' perspectives on the first year of the COVID‐19 pandemic. In summary, the informants thought that care homes should primarily provide a home‐like environment although a facility design that enables isolating residents including those with dementia was considered beneficial. Some also suggested more possibilities for staff to get changed and store PPE as they enter/leave each resident's apartment. The informants further stressed that experienced employees are invaluable when facing an infectious outbreak. A mix of permanent and temporary staff was considered essential for the care home service to operate properly, although some believed that temporary staff might also negatively influence the spread of infection. Language barriers were highlighted as an obstacle when trying to disseminate information about COVID‐19.

Informants valued the home‐like environment, yet thought that it would be beneficial to also have areas equivalent to ante‐rooms in hospitals which allow for storage of PPE and staff to get changed as they enter/leave each resident's apartment. Previous research on ante‐rooms in care homes is sparse, primarily focusing on air circulation (Drinka & Krause, 2004; Miller et al., 2021; Wang, 2020), and little attention has been paid to its potential to also store PPE (Lee & Jeong, 2020; Wang, 2020). Recently, compared to age‐related declines of the residents, the built environment of care homes has been considered modifiable (Wang, 2020), and ante‐rooms in care homes for older adults could be multifunctional in facilitating infection control (Lee & Jeong, 2020; Wang, 2020). In Sweden and elsewhere, the purpose of care homes including the balance between being able to provide a home‐like atmosphere and healthcare is now discussed (Miller et al., 2021; Schön & Fritzell, 2021; Szczerbińska, 2020). The importance of involving staff such as nurses in the development of policy and regulations has been stressed in order to make future policies inclusive and sustainable (Morley et al., 2020). These arguments are also provided in a recent study emphasising advantages of involving nurses when designing care facilities (Miedema et al., 2022).

Most informants thought that having a smaller number of residents is positive in terms of risk of a COVID‐19 outbreak. While previous research has demonstrated an association between crowded care homes and infectious outbreaks (Leece et al., 2023), residents in Swedish care homes do not share bedrooms and can have dinner in their apartment during an infectious outbreak. Therefore, the findings on smaller number of residents to prevent and control infection seem to be more relevant to discuss in relation to facility size rather than population density. A growing body of evidence suggests that larger care homes with a larger number of residents are associated with increased risks of COVID‐19 outbreaks (Abrams et al., 2020; Morciano et al., 2021; Shallcross et al., 2021; Smittskydd Stockholm., 2020; Stall et al., 2020). Yet larger facilities do not necessarily face large outbreaks, possibly thanks to greater financial resources allowing for purpose‐built facilities and more staff resulting in greater ability to isolate infected sections and less cross‐over of staff between residents (Halloran et al., 2020; Stall et al., 2020). Indeed, some informants stressed that cohort care is essential for infection control and a previous study has shown that reducing the number of residents per unit from 20 to 10 has a positive impact on cohort care (Nguyen et al., 2021). In the present study, cohort care was, however, challenged when having residents with dementia who wander, a finding that supports existing literature showing that handling residents who have a strong compulsion to walk has been particularly challenging when practising isolation and social distancing (Liddell et al., 2021). Furthermore, the informants thought that a larger workforce facilitates cohort care and some of them argued that employees hired by the hour allow for a higher number of employees in total. Most informants did not think that having a high number of temporary employees increases the risk of spread of infection, although some informants were of a different opinion. Studies on the potential impact of temporarily hired staff on infectious disease outbreaks seem to vary as one study has reported the risk of COVID‐19 among residents to be greater in care homes that had temporary staff only a few times per month compared to having temporary staff more regularly or having no temporary staff (Shallcross et al., 2021). However, other studies have reported no association between use of temporary staff and COVID‐19 outbreaks (Bowblis & Applebaum, 2021; Rolland et al., 2020). In the present study, informants' views on a potential relationship between temporarily hired staff and infectious outbreaks might have been influenced by the fact that some were dependent on temporarily hired staff. Following the severe acute respiratory disease (SARS) in 2003, the dependency on temporary staff in the care sector was discussed and research concluded that the staffing situation had left the healthcare system vulnerable and without spare capacity to handle a new crisis similar to the SARS epidemic (Baumann et al., 2006). The same scientific article also concluded that it was fortune that SARS did not spread more widely in the community (Baumann et al., 2006). Having employees hired by the hour is furthermore likely to primarily be of interest to the employer as research has shown negative outcomes to such employees in the form of job insecurity negatively affecting the individual's health and well‐being (Moscone et al., 2016). Besides, job insecurity in nursing could be considered particularly serious as it may affect both patients and their family members in the form of possible patient safety incidents, and society in terms of poorly functioning care systems.

All informants recalled particularly the beginning of the pandemic as being challenged by absenteeism due to sickness and fear of infection. Unsurprisingly, having experienced staff on‐site during an exceptional event such as a COVID outbreak was considered invaluable. Continuous studies have reported qualified and experienced care home staff to be essential to meet the often complex care needs of older people residing in care homes (Devi et al., 2021; Wild et al., 2010). Yet, in many countries, attracting, recruiting and retaining care home staff is an ongoing challenge due to precarious work conditions, poor social status and lower pay levels compared to jobs with similar qualifications in other health sectors (OECD, 2020b). Additionally, the informants considered speaking little Swedish to be an obstacle when trying to disseminate information about the coronavirus. In Sweden, many care home nursing aides are of foreign background and speak little Swedish (Gunnarsson & Klingberg Hjort, 2018; OECD, 2020a). Yet, research has shown that addressing nursing aides' language and communication skills has multiple benefits including reduced stress levels (Sprangers et al., 2015). The potential consequences of nursing aides with limited skills in the Swedish language should be considered particularly important as poor language proficiency among foreign‐born people not only impedes access to information on COVID‐19 (OECD, 2020a) but also increases the risks of misunderstandings (Gunnarsson & Klingberg Hjort, 2018), possibly jeopardising infection control.

Implications include arranging for storage and changing facilities of personal protective equipment possibly in the form of temporary or permanent ante‐rooms as one enters/leaves a resident's apartment. It is further important that care home workers are trained and get to practice and develop their skills as experienced staff is considered invaluable by care home managers during infectious outbreaks. Minimising the number of different care homes temporary staff work might also positively reduce the risk of spread of infection. Preparing how to organise the work in the event of an infectious outbreak and information on infection control in different languages and formats (not just in written) for fast provision and action could furthermore facilitate the handling of an infectious outbreak.

### 4.2 Strengths and limitations

Study strengths include that it was conducted during the first year of the COVID‐19 pandemic, reducing the risks of recall bias. The study's focus on the impact of facility and staff characteristics on COVID‐19 infection control in care homes for older adults further adds to research on a topic less studied. While findings in the interview material repeatedly reoccurred, the small number of informants may affect the possibility of ensuring data saturation and consistency and drawing firm conclusions. The possible lack of data saturation might mean that some diversity, depth and nuances of the subject studied have been missed (O'Reilly & Parker, 2012). However, according to the concept of information power in qualitative research, even a small number of participants could be considered sufficient if the research topic is almost unexplored (Malterud et al., 2016). The care homes were further purposively selected, enabling ‘depth’ but making it difficult to replicate the study. Yet, the sampling strategy allowed for informants to represent public, private, small, medium and large care homes for older adults, enhancing credibility of the study (Zhang et al., 2011). Nevertheless, the informants' perspectives are influenced by their management responsibilities and their financial budget. For example, the informants were in favour of the care home visiting ban and use of temporary staff; aspects of care homes that have been heavily debated and divided by the general public and politicians yet considered essential by the informants. This one‐sided perspective is important to keep in mind as care professionals' perceptions differ depending on their position in the healthcare occupational hierarchy (Zhang et al., 2011). Study limitations also include the risk of recall bias when asking informants about an event that began a year before the data collection. Furthermore, conducting interviews by video call and telephone allowed for flexibility in terms of location and was necessary due to the ongoing pandemic. Yet, it restricts any observations of behaviour and body language, and the informant might feel less comfortable in expressing their true experience without being judged. All interviews were, however, conducted by the same researcher in less than a month, reducing the risk of unintended differences in how the interviews were undertaken and strengthening the study's dependability (Graneheim & Lundman, 2004).

## 5 CONCLUSIONS AND FURTHER RESEARCH

In conclusion, care home managers expressed that facility design that enables infection control and experienced staff facilitate the handling of infectious disease outbreaks such as the COVID‐19 pandemic. Care home managers further preferred home‐like environment over a more clinical environment yet thought that the current lack of designated areas for storage and change of PPE directly following a visit of an (potentially) infected resident should be addressed to improve infection control. Further research should capture the views of care home employees, including nurses and nursing aides, and residents to further identify characteristics of facility design that might facilitate infection control. Multidisciplinary intervention studies to improve infection control through changes to the care home facility design could furthermore contribute to better handling of future pandemics similar to COVID‐19.

## AUTHOR CONTRIBUTIONS

LM, AL and BB designed the study. LM and AL conducted the study with input from JA. AL and LM wrote the manuscript and BB, PS and JA critically revised it. All authors have read and approved the submitted version.

## FUNDING INFORMATION

The study is funded by the Swedish Research Council (2020–05850).

## CONFLICT OF INTEREST STATEMENT

The authors declare no conflicts of interest.

## ETHICS STATEMENT

The study has received research ethics committee approval by the Swedish Ethical Review Authority (2020–04577).

## Supporting information


Data S1.

Data S2.

## Data Availability

Data available on request from the authors.

## APPENDIX A Topic guide

### A.1

To begin with, I will ask a few questions to get a better picture of the care home.

1. How many residents do you have?
2. Is the care home divided into units? If yes, how many units?
  - What type of units? (dementia, somatic)
  - How many residents per unit?
  - To what extent are the units connected?
  - What activities take place in each unit? (Communal areas?)
3. Does the care home have multiple floors?

Staff‐related questions

4 Roughly how many employees do you have?
5 Do employees work at more than one unit?
  - Do night‐shift staff work at more than one unit?
6 Roughly what proportion of staff are on permanent contracts?
  - What proportion of staff work full‐time/part‐time?
  - Do you know if staff have secondary employment?
7 What proportion of staff are on short‐term contracts?
8 What levels of competencies do staff have?

COVID‐19‐related questions

9 Please describe what actions have been taken to protect the residents?

COVID‐19 in relation to the care home's size and physical buildings

10 Has the care home's physical building(s) influenced how you've handled the pandemic (irrespective of preparation of/actual infectious outbreak)?
  - Have the physical buildings hindered action?
  - Anything related to the buildings, indoors and outdoors, that has facilitated action?
  - Anything in the physical buildings that has been of particular relevance (as an advantage or disadvantage) in terms of infection control and caring for residents with dementia?
11 What importance does the physical building have in preventing and reducing spread of infection, do *you think*?
12 Did the buildings allow for cohorting?
13 In terms of the physical buildings, what do *you consider* to be different in infection prevention in a care home compared to a hospital?
14 In what ways *do you think* the size of the care home might matter for infection control?

Staffing levels in care homes during the pandemic

15 Do you think that the staffing levels affected your work on infection prevention/control?
16 To what extent has the care home been affected by staff absenteeism during the pandemic?
17 Was your work on infection prevention/control affected by staff's form of employment?
18 What are your thoughts on the possibility that large proportions of staff on short‐term contracts increase the risks of infectious outbreaks?
19 In your experience, do all care workers have the same level of knowledge and understanding of infection control work?
20 Have language barriers hindered infection control?

Closing questions

21 In hindsight, what actions taken have been shown to be useful for infection control?
22 In hindsight, would some actions have been taken that for various reasons were not taken?
23 How would you design care homes to facilitate infection prevention and control?
24 Anything that you would like to add?
